# Recurrent embolic strokes due to antiphospholipid syndrome and non-bacterial thrombotic endocarditis in a patient with basal cell carcinoma

**DOI:** 10.1186/s13019-023-02266-6

**Published:** 2023-05-11

**Authors:** Julianna Svantner, Luc Lavanchy, Ania Labouchère

**Affiliations:** 1 Department of Intensive Care, Hospital of Valais, Sion, Switzerland; 2 Department of Anesthesiology, Lausanne University Hospital and University of Lausanne, Lausanne, Switzerland; 3 Department of Plastic and Hand Surgery, Lausanne University Hospital and University of Lausanne, Lausanne, Switzerland

**Keywords:** Antiphospholipid syndrome, Non-bacterial thrombotic endocarditis, Heart valve disease, Valve replacement surgery, Stroke, Thrombosis

## Abstract

**Background:**

Non-Bacterial Thrombotic Endocarditis (NBTE) is a common form of aseptic thrombotic endocarditis that primarily affects mitral valves and less frequently aortic valves. NBTE is caused by systemic inflammatory reactions. This condition induces valve thickening or attached sterile mobile vegetation. NBTE is mostly asymptomatic; however, major clinical manifestations result from systemic emboli rather than valve dysfunction. When significant damage occurs, valvular insufficiency or stenosis can appear and promote heart failure occasionally requiring valve replacement surgery. NBTE is associated with hypercoagulable states, systemic lupus erythematous (SLE), antiphospholipid syndrome, or malignancies.

**Case presentation:**

We report successful biological aortic valve replacement surgery including cardiopulmonary bypass for a 78-year-old man with NBTE and voluminous vegetation on the aortic valve inducing moderate aortic insufficiency and acute heart failure. The histopathological analysis of the valve sample showed myxoid degeneration, fibrous remodeling, and partial necrosis without any bacteria, thus indicating marantic endocarditis. Initially, the patient presented to the emergency department with an acute stroke. He was already using Apixaban treatment for a history of atrial fibrillation and cardioembolic stroke. Because of the recurrence of stroke and appearance of aortic vegetation, the investigations were extended. The antiphospholipid antibodies were positive without any indication of bacterial endocarditis. The malignancy screening was positive for basal cell carcinoma (BCC). The surgery and postoperative course were uneventful, and the patient was discharged with vitamin K antagonists (VKA). To our knowledge, NBTE with such a volume is rare and its apparent association with BCC has not been previously reported.

**Conclusion:**

Outside of SLE and antiphospholipid syndrome, NBTE is a rare and underdiagnosed disease associated with thromboembolic events. Adequate anticoagulation is a cornerstone of its treatment. Anticoagulation management during perioperative care and valve surgery deserves specific attention and helps to protect the patient from embolic complications. In the case of stroke and thromboembolic events of unclear cause or suspected NBTE, echocardiography and thrombophilia assessments including an immunological workup are recommended.

## Background

Non-Bacterial Thrombotic Endocarditis (NBTE), also known as marantic endocarditis, outside of autoimmune disease and malignancies, is a rare disease with a prevalence of 0.9–1.6% that was described by Ziegler in 1888 as thromboendocarditis [1]. NBTE encompasses thrombotic, inflammatory, and inflammatory with superimposed thrombotic vegetations. It is a cardiac manifestation of an underlying inflammatory process that induces endothelial lesions of the valve and endocardium. The mitral valve is the most frequently affected, followed by the aortic valve. Histologically, the lesions consist of an accumulation of immune complexes, fibrin, platelet thrombi, and mononuclear cells. The typical presentation of NBTE is valve thickening or sterile vegetation ranging from small nodules to a large warty or necrotic mass. This disease is mostly asymptomatic. Clinical manifestations mainly result from systemic arterial embolism such as stroke or transient ischemic attack. Valve dysfunction is uncommon and is usually secondary to the size and location of the endocardiac mass without significant destruction. NBTE may be associated with a hypercoagulable state or autoimmune conditions such as systemic lupus erythematous (SLE), antiphospholipid syndrome (APS), rheumatoid arthritis, or malignancies [2]. It is well known that in patients with APS, valvular manifestations are very common as well as their strict association with cerebral manifestations [3]. We report a rare fatal case of a voluminous aortic NBTE in a patient who developed multiple embolic strokes caused by antiphospholipids antibodies (aPL). This occurred in a 78-year-old man using Apixaban for a history of atrial fibrillation. He presented with moderate aortic insufficiency, acute heart failure and was successfully treated by biological aortic valve replacement surgery.

## Case presentation

In August 2021, a 78-year-old Caucasian man using Apixaban presented to the emergency department with mild aphasia associated with balance disorders for 36 h. The direct-acting oral anticoagulant (DOAC), which was an anti-Xa inhibitor, had been implemented many years ago because of a history of paroxysmal atrial fibrillation. The patient stated that he used Rivaroxaban until November 2019. At that time, anticoagulation had to be briefly stopped so he could undergo elective resection of a perineal lipoma. The morning after stopping treatment, the patient woke with dysarthria, left labial ptosis, weakness of the left arm, and clumsiness. Computed angiotomography of the brain showed a right opercular cardioembolic stroke. Brain magnetic resonance imaging (MRI) demonstrated a right sylvian multiembolic stroke with right opercular and insular lesions. Because this first infarction was possibly promoted by suboptimal anticoagulation, Rivaroxaban was changed to Apixaban without further investigations. The patient showed complete neurological recovery.

On admission in August 2021, the patient was hemodynamically stable with a regular cardiac sinus rhythm. Neurological examination findings included production aphasia and discrete ataxia.

Routine blood test results showed a slight decrease in the platelet count (90 × 10^6^ g/L) and prolonged activated partial thromboplastin time (44 s) [4]. The inflammatory parameters were in the normal range.

**Fig. 1:**
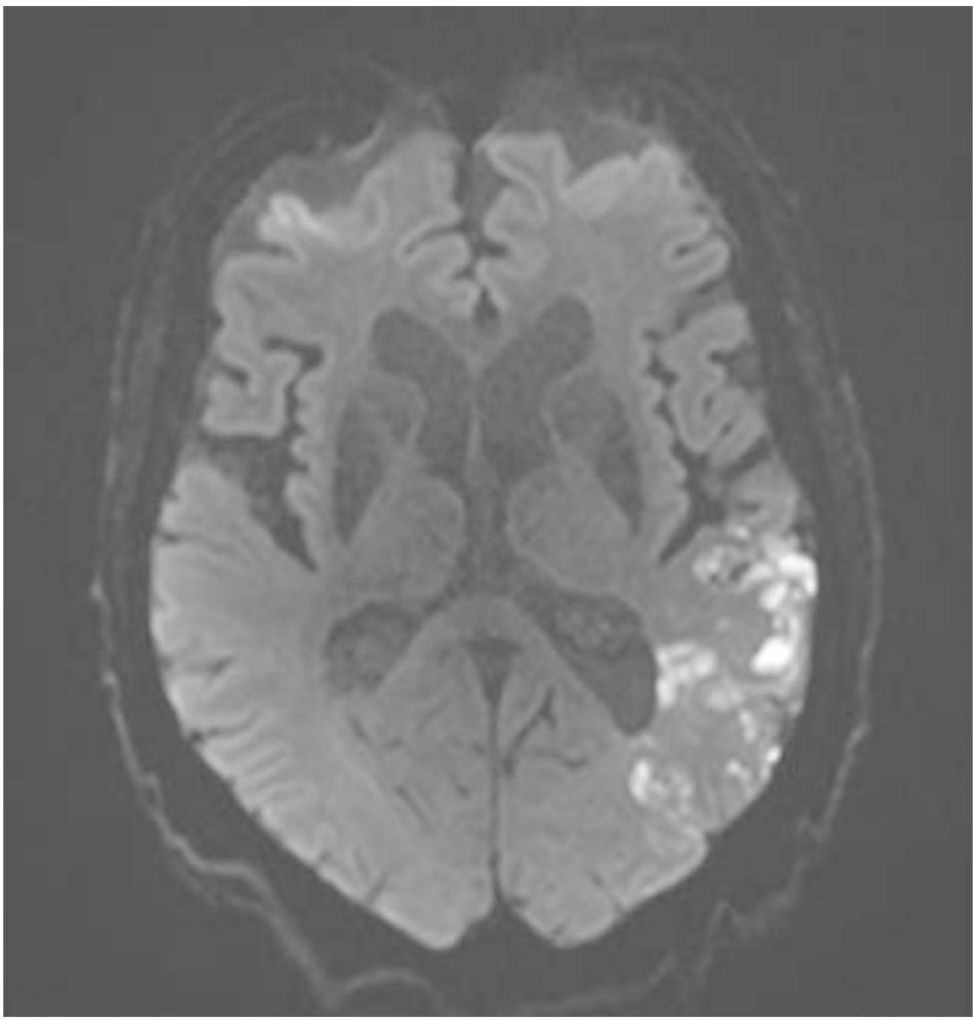
Magnetic Resonance Imaging (MRI) of the brain illustrating ischemic lesions in the territory of left middle cerebral artery suggestive of cardio-embolic origin

**Fig. 2:**
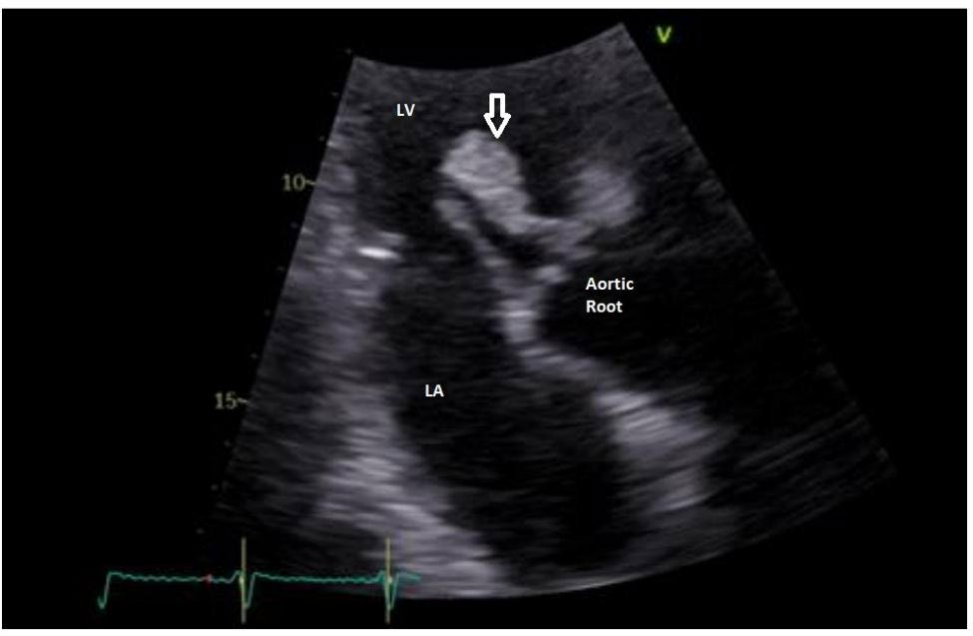
Transthoracic echocardiography (TTE), three-chamber zoomed view, illustrating a voluminous aortic vegetation (arrow) attached to the ventricular side of the right coronary aortic cusp

Computed angiotomography of the brain did not reveal any new stroke. The brain MRI, which was performed the day after admission, showed an acute left sylvian stroke (Fig. 1). Because of the new cerebrovascular event occurring with efficient anticoagulation, further investigations were warranted. Transthoracic echocardiography showed a fluctuating mobile pedicle mass (31 × 14 mm) attached to the aortic valve [5] (Fig. 2) and inducing moderate aortic insufficiency (grade 2 to 3/4). The hemato-immunological results were as follows: Lupus antibodies were negative for SLE [Antinuclear antibodies – ANA (< 160 1/DIL): ***> 640 1/DIL*** (indirect immunofluorescence assay – high sensitivity, low specificity). Anti-dsDNA, anti-Smith, anti-Ro/SSA, anti-La/SSB, anti-U1RNP and anti-histones: all negative (enzyme linked immunosorbent assays (ELISA) - low sensitivity, high specificity)] [6]; aCL (Anticardiolipin antibody) (IgG < 20 U): ***1 506 U***; anti-β2GPI (Anti-beta2 glycoprotein 1 antibody) (IgM < 20 U): ***34 U***; anti-β2GPI (IgG < 50 U): ***> 6 100 U*** (ELISA) - signing a moderate to high risk of APS [7]. Blood culture and bacterial serology results were sterile (*Coxiellea burnetti*, *Brucella sp, Bartonella sp*, *Legionella*).

**Fig. 3:**
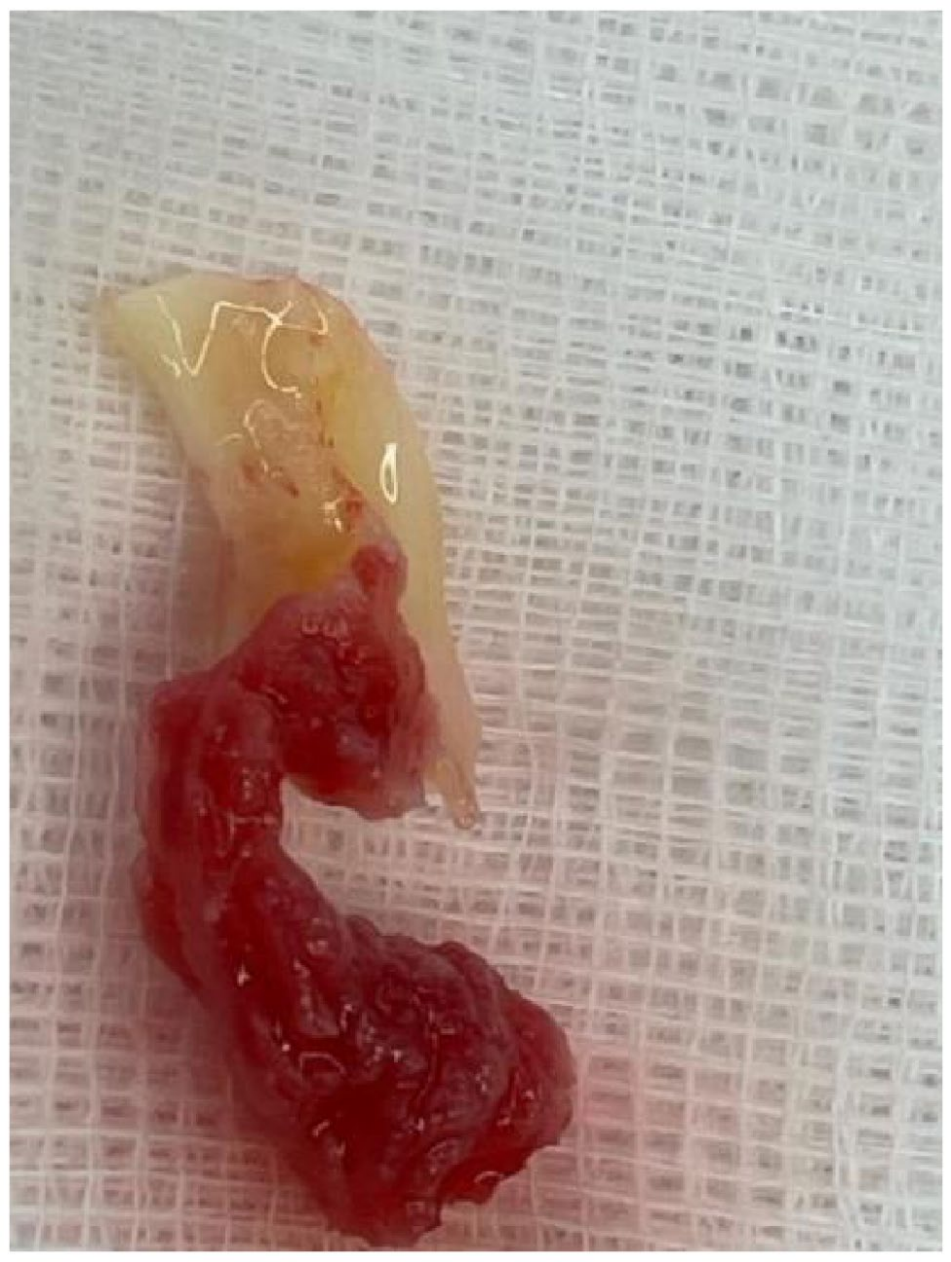
Excised aortic valve cusp shows an attached pink gelatinous pedicle vegetation (4 × 1.5 cm) adherent to the atrial surface of the right coronary leaflet

**Fig. 4:**
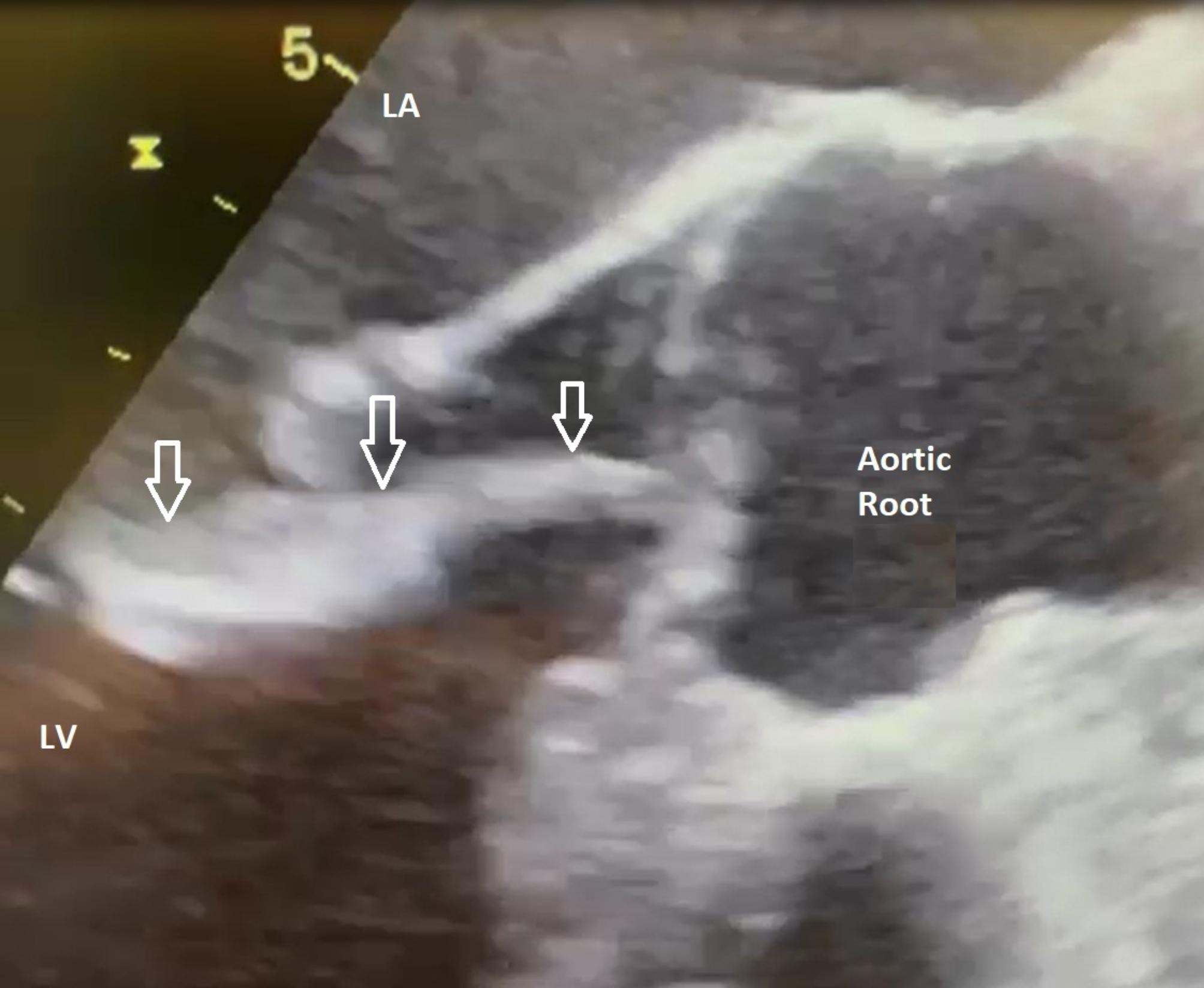
Two dimensional transoesophageal echocardiography (TEE), midesophageal aortic valve long axis view, revealed fluctuating mobile pedicle vegetation attached to the free edge of the right coronary leaflet of the aortic valve

Because of the large size of this intracardiac mass and the related occurrence of embolic events, short-term valvular surgery was indicated [8]. The cardiac surgery team proceeded with biological aortic valve replacement. Exposure of the aortic valve revealed a tricuspid valve of normal morphology except for a prolapsed noncoronary leaflet creating the insufficiency. The vegetation was gelatinous and had an elongated teardrop shape, with an approximate length of 4 cm and a maximum width of 1.5 cm that was attached to the free edge of the right coronary leaflet (Fig. 3). A small amount of similar material was also found on the left leaflet. The valve could not be preserved because of insufficient coaptation of the noncoronary leaflet and strong adherence of the mass to the cusp surface. After excision of the leaflets, a biologic aortic valve prosthesis, Edwards Inspiris Resilia 27 mm ® was implanted. The intraoperative transesophageal echocardiography image of the vegetation is illustraded on Fig. 4. International guidelines for APS therapy do not recommend to use Apixaban. Therfore the postoperative heparin was bridged with vitamin K antagonists (VKA) – Acenocumarol[7]. The histological examination revealed a fibrin cluster without acute inflammation that suggested marantic endocarditis.

Subsequent echocardiography confirmed normal functioning of the aortic prosthesis, and the patient was released from the hospital without any postsurgical complications. The aphasia resolved and, at the time of re-evaluation, the neurological deficits were absent.

**Fig. 5:**
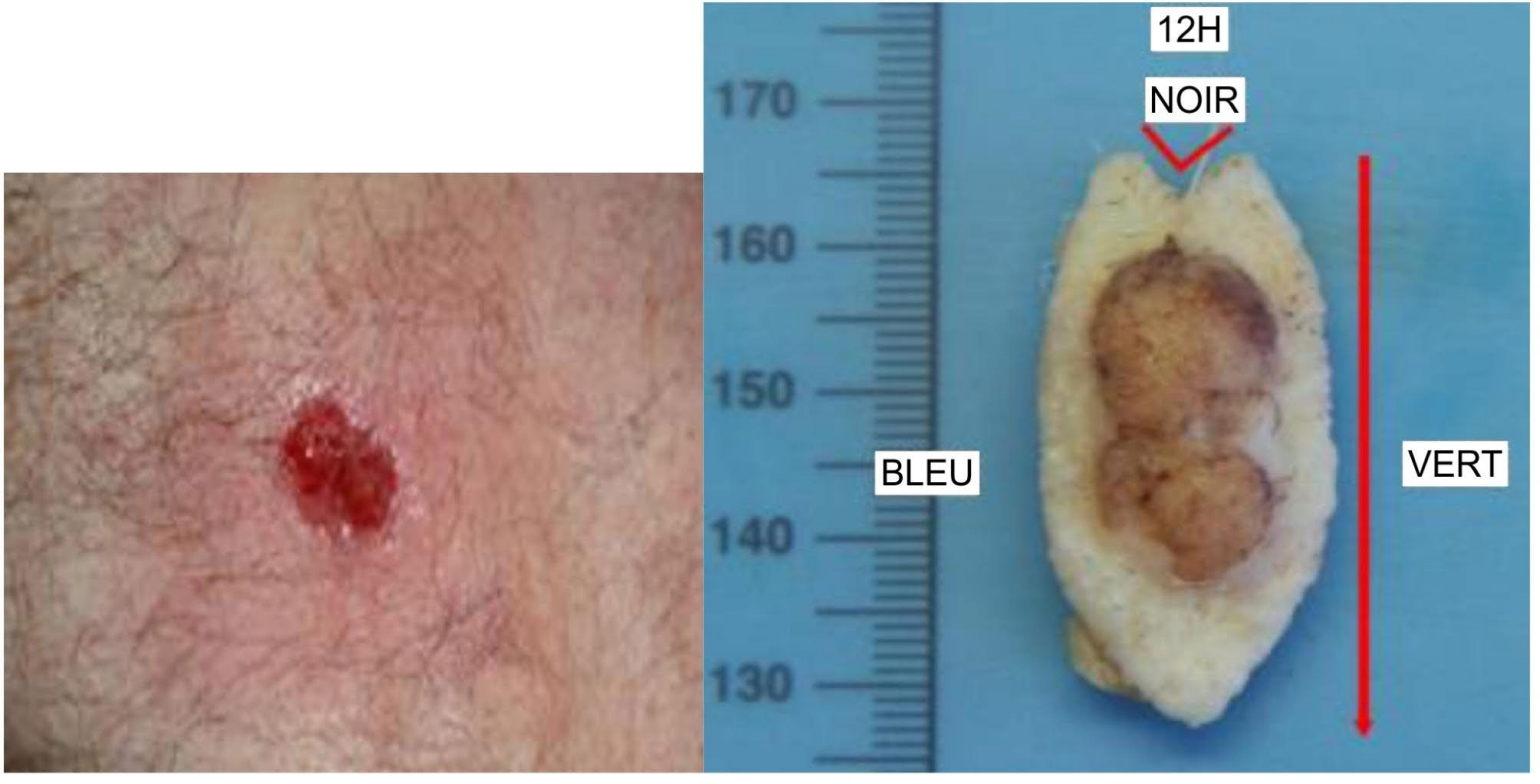
BCC of solid type located on the sternum, before and after excision

**Fig. 6:**
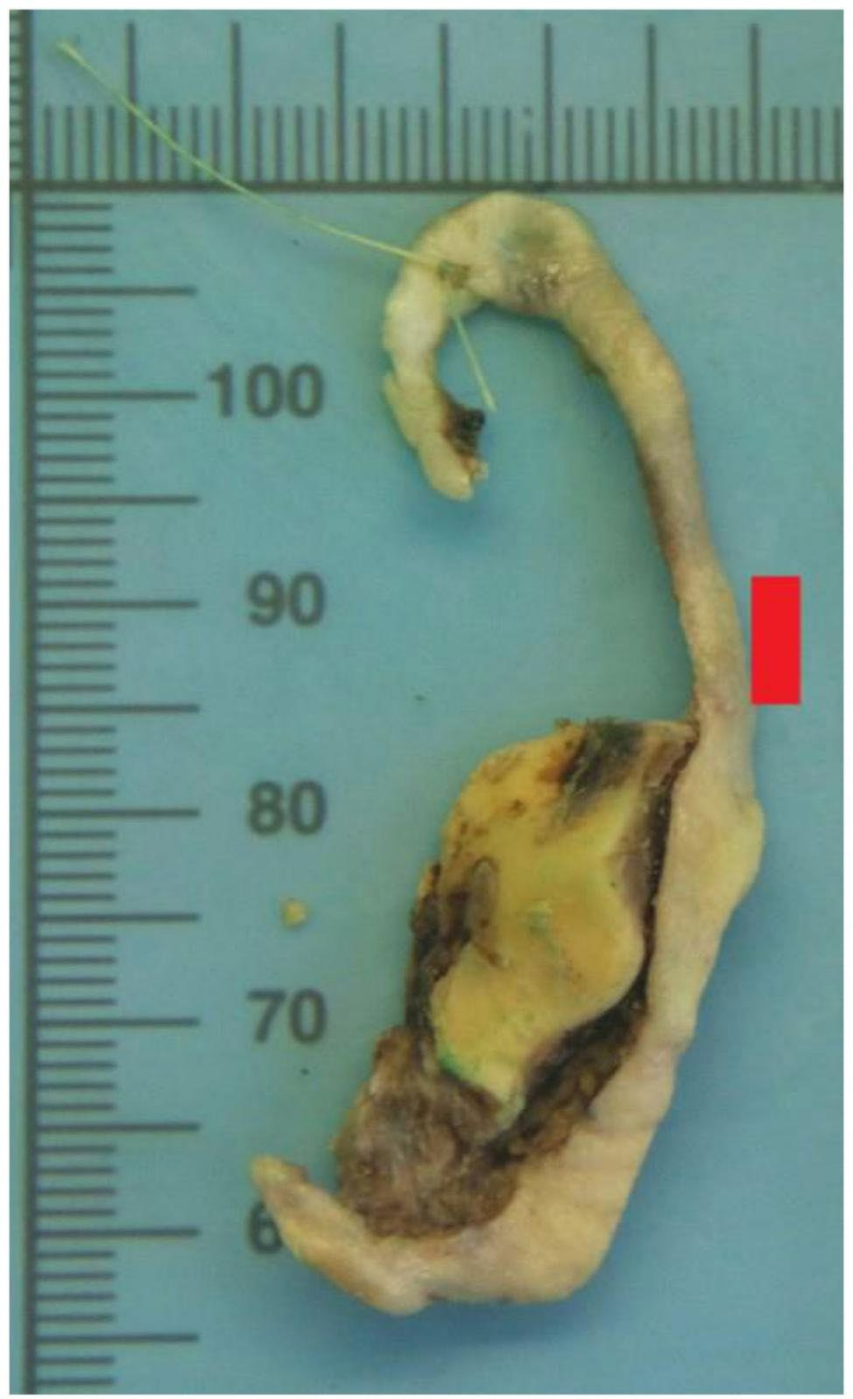
BCC (5.5 × 1.5 × 0.7 cm) located inside of the left ear, after excision

A few weeks before admission, a bulging skin lesion appeared on the sternum (Fig. 5). It was removed in January 2022, at 5 months after heart surgery, and was observed to be solid BCC. In March 2022, residual BCC of the ear was identified and removed (Fig. 6). At 6 months postoperatively, there was no clinical thromboembolic recurrence.

## Discussion

Our report demonstrates how recurrence of cerebral infarctions in a patient using long-term anticoagulation can lead to a diagnosis of aortic NBTE and APS with positive aCL and anti-β2GPI antibodies. This uncommon condition was successfully treated by surgery, treatment of underlying oncological disease, and dedicated anticoagulation.

**Fig. 7:**
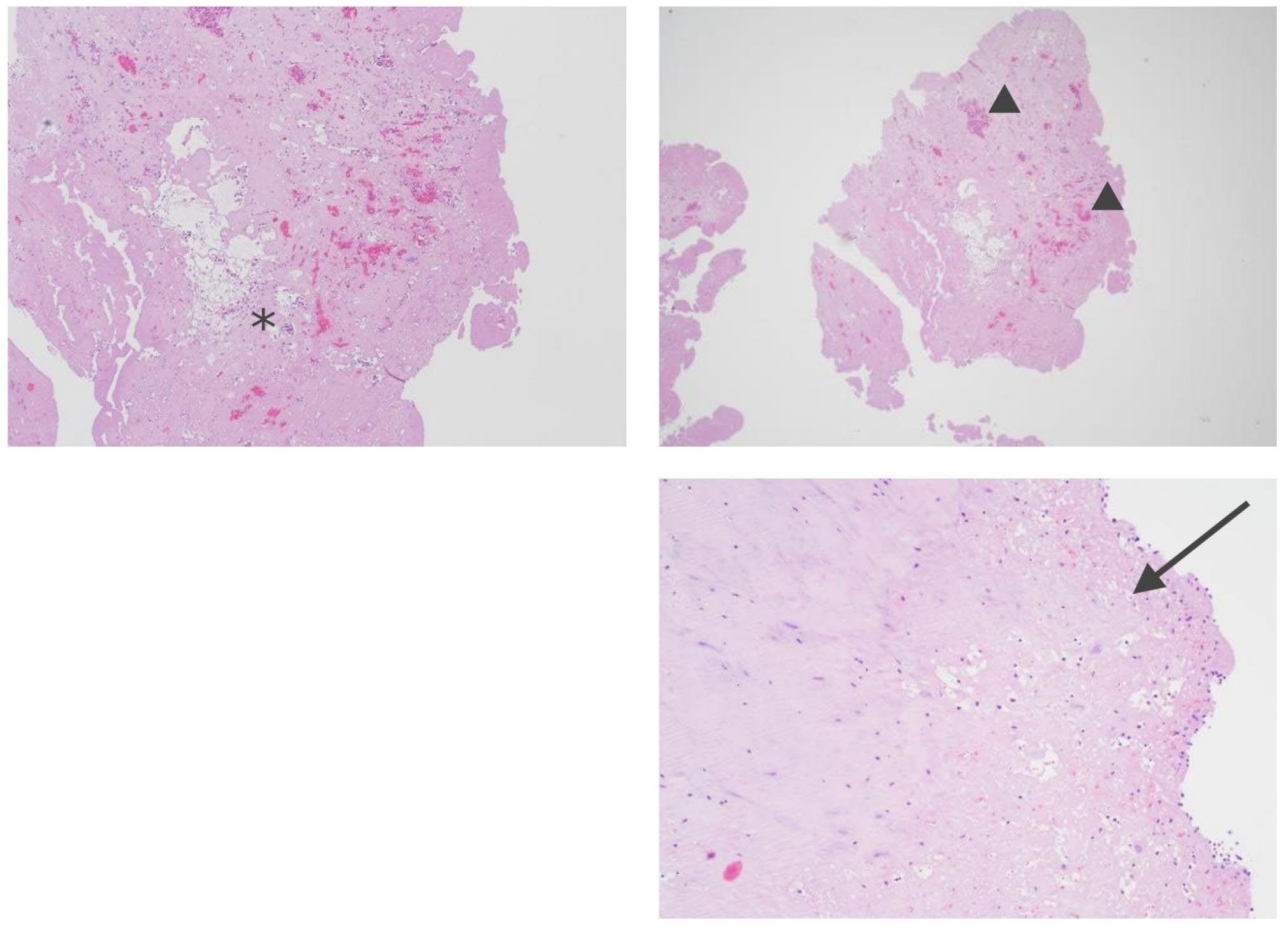
Histological sections of vegetation: illustrating a myxoid degeneration (arrow), fibrous remodeling (arrowhead) and partial necrosis (asterix), without any germs

For our case, the diagnosis of NBTE was confirmed after surgical removal of the mass and its histopathological examination (Fig. 7). So far, the exact pathogenesis of NBTE is unclear. It is agreed that vegetations are the result of an underlying systematic inflammatory process that develops after endothelial injury. This process occurs in the setting of a hypercoagulable state, such as SLE, APS, or malignancies [9]. The vegetation varies from microscopic endothelial lesions to large friable structures that can lead to cerebral and systemic embolisms [10]. The diagnosis is based on high clinical suspicion, and it is confirmed by a pathological analysis of the surgical sample. The vegetations may be found anywhere on the endocardium, but they are most commonly observed on the left side of the heart and attached to the upstream side of the valve leaflets [11, 12]. The mitral valve is the most frequently involved, followed by the aortic valve; their damage can induce functional regurgitation. In addition to embolic events, NBTE clinical presentation can include asymptomatic valvular thickening, heart failure caused by valvular dysfunction, and unspecific symptoms such as fever, arthritis, a new murmur, splinter hemorrhages, fatigue, night sweats, and weight loss [2, 13]. Old people can be diagnosed as APS but it is considered as a diagnosis of exception in patients over 60 year old. This is constantly observed in this population since the disease is rare and there are few specialized centers around the world.

Identifying the underlying inflammatory process leading to NBTE and its reversal is the mainstay of treatment. Therefore, the immunohematological workup is essential. It is known that APS may manifest itself as primary or secondary disease, most commonly in the context of SLE with various neurological and cardiac manifestations [14, 15]. Investigations of APS include determining the dose of lupus anticoagulants (LA) and aPL such as aCL and anti-β2GPI [2, 7, 16]. Contrary to LA, aPL are unaffected by Apixaban, and they are the only ones to be screened in patients with ongoing anticoagulation[17]. Diagnostic criteria for APS are based on the Sapporo classification criteria (Table 1) [2]. The clinical expression of APS includes thrombotic or obstetric events. Thrombotic events can be arterial, such as transient ischemic attack, stroke, and limb ischemia, or venous, such as deep venous thrombosis and pulmonary embolism. Obstetric complications can appear before 10 weeks of gestation as three or more unexplained consecutive spontaneous abortions, or as unexplained deaths of a morphologically normal fetus after 10th week of gestation as well as premature birth of a morphologically normal neonate before the 34th week of gestation.

**Table 1:** Sapporo classification criteria [2]

Clinical criteria
1. **Vascular thrombosis**
≥1 Clinical episodes of arterial, venous, or small vessel thrombosis
2. **Pregency morbidity**
≥1 Unexplained deaths of a morphologically normal fetus ≥ 10th week of gestation
≥1 Premature births of a morphologically normal neonate < 34th week of gestation due to: - eclampsia - severe pre-eclampsia - placental insufficiency
≥3 Unexplained consecutive spontaneous abortions < 10th week of gestation (with exclusion of maternal anatomic, hormonal or chromosomal abnormalities)
**Laboratory criteria**: on 2 or more occasions at least 12 weeks apart
LA present in plasma
aCL of IgG and/or IgM isotope in serum or plasma, present in medium or high titer (> 40 GPL or MPL, or > the 99th percentile)
Anti-β2GP1 of IgG and/or IgM isotope in serum or plasma (in titer > the 99th percentile)

Other clinical manifestations that are not included in the Saporro classification are thrombocytopenia, hemolytic anemia, acute thrombotic microangiopathy, valve vegetation or thickening, livedo reticularis, cognitive dysfunction, and subcortical white matter changes [4].

The role of the aPL in the pathogenesis of NBTE is unclear. Some authors suggested that they may induce an autoimmune reaction against endothelial membranes, thus inducing fibrin-platelet thrombi deposits [18]. Endothelial damage of the valvular surface caused by microinjury is another presumed mechanism that can lead to fibrin-platelet formation [19]. Further organization of these lesions in addition to immunoglobulins and complement component deposits lead to thrombosis, fibrosis, and thickening of the cusps [20].

The interpretation of APS using laboratory testing can be classified using three profiles (Table 2) [4, 7]. In our case, because of ongoing Apixaban treatment, and according to the recommendations of the International Society on Thrombosis and Haemostasis (ISTH), LA could not be searched. The DOACs can cause false-positive LA results [16, 17, 21]. Despite the lack of LA testing, and based on the interpretation of aPL, we concluded that the patient had at least medium-high aPL titres and was at moderate to high risk for APS. Considering the high risk profile of this patient it was assumed there was a high probability that he could be triple positive, what definitely increases his thrombotic risk. According to European Alliance of Associations for Rheumatology recommendations, we aimed to perform the second immunological workup after 12 weeks [4, 7].

**Table 2:** Definitions of medium-high aPL titres, and of high-risk and low-risk aPL profile [7]

Medium-high aPL titres • aCL of IgG and/or IgM isotype in serum or plasma present in titres > 40 IgG phospholipid units or > 40 IgM phospholipid units, or > the 99th percentile, measured by a standardised ELISA. anti-β2GPI of IgG and/or IgM isotype in serum or plasma in titre > 99th percentile, messured by standardised ELISA.
**High-risk aPL profile** • The presence (in 2 or more occasions at least 12 weeks apart) of LA (measured according to ISTH guidelines), or of double (any combination of LA, aCL, or anti-β2GPI) or triple (all three subtypes) aPL positivity, or the presence of persistently high aPL titres.
**Low-risk aPL profile** • Isolated aCL or anti-β2GPI at low-medium titres, particularly if transiently positive.

Given that the serological screening for SLE were negative, the clinical suspicion was low.

In our case, the malignancy screening results were positive. The patient presented with solid BCC located on the skin of the sternum near the heart. According to the literature, the most frequent malignancies associated with APS include solid tumors of the lung, colon, cervix, prostate, kidney, ovary, breast, and bone, Hodgkin disease, non-Hodgkin lymphoma, myeloid leukemia, and lymphocytic leukemia [22, 23]. At the time of discharge, no further tumor screen work up was done. BCC are common in 78 year old Caucasians, this diagnosis is likely incidental and not causative. We aimed to perform the second immunological workup after the complete the removal of BCC [24]. This might suggest that the patient is suffering from primary APS.

Similar to infective endocarditis, hemodynamically significant valvular lesions and thromboembolic phenomena are indications for valvular surgery [25], which is associated with higher mortality and morbidity for patients with APS [26]. In our case, because of embolism recurrence and the large vegetation, the benefits overcame the risks, and the surgical team decided to proceed with surgery. Usually the treatment for NBTE is anticoagulation, unless the patient is hemodially unstable, in which case surgery is required. The treatment of APS and, consequently, NBTE depends mainly on the clinical presentation. Primary thrombosis prevention with aspirin should be considered for obstetric clinical APS cases or when aPL are positive without any associated thromboembolic event. VKA are recommended for secondary prevention of thrombosis, with a minimal target international normalized ratio of 2–3. Low-molecular-weight heparin is recommended for thrombosis prevention during high-risk periods, such as the perioperative or peripartum period [4, 7]. Hydroxychloroquine can be added in the case of thrombosis recurrence despite therapeutic anticoagulation. Glucocorticoids, intravenous immune globulins, or plasma exchange are recommended only in case of catastrophic APS [4, 27]. DOACs are less effective than VKA in the treatement of APS since reccurences of arterial thrombosis have been reported [4, 7, 28–31]. When the diagnoses of NBTE and APS have been confirmed, in accordance with the 2015 European Society of Cardiology and the European Alliance of Associations for Rheumatology guidelines, anticoagulation therapy including VKA with a target international normalized ratio of 2 to 3 was administered [4, 7, 16, 17, 25].

## Conclusions

Patients presenting with an idiopathic stroke and a high suspicion of underlying immunological, hematological, or oncological disease should undergo testing for possible nonbacterial endocarditis and underlying autoimmune diseases. Transthoracic echocardiogram images should be obtained to determine if lesions are present. Transesophageal examination is the best dedicated diagnostic tool because it helps to avoid missed diagnoses. Treatment of the underlying condition, resection of the vegetation if indicated, the introduction of dedicated anticoagulation, and careful follow-up are important to the successful treatment of this uncommon condition. Moreover patients with underlying evidence of ischemic insult, especially if younger than 60 years old, without major thrombotic risk factors, should be evaluated for the presence of aPL in order to evaluate the antecipation of an APS diagnosis. Patients who have non-criteria APS manifestations such as thrombocytopenia, should be screened for valvopathy, since 30% of APS patients have a silent cardiac disease that is frequently missed.

## Data Availability

Not applicable.

## List of abbreviations

NBTE: Non-bacterial thrombotic endocarditis
SLE: Systemic lupus erythematous
APS: Antiphospholipid syndrome
aPL: Antiphospholipids antibodies
DOAC: Direct-acting oral anticoagulant
MRI: Magnetic resonance imaging
ELISA: Enzyme linked immunosorbent assays
ANA: Antinuclear antibodies
aCL: Anticardiolipin antibody
anti-β2GPI: Anti-beta2 glycoprotein 1 antibody
LA: Lupus anticoagulants
ISTH: International Society on Thrombosis and Haemostasis
BCC: Basal cell carcinoma
VKA: Vitamin K antagonists
